# The Risk of Aspiration Is Low With Continuing Semaglutide During Elective Eye Surgery When Patients Receive Only Moderate Sedation

**DOI:** 10.7759/cureus.102921

**Published:** 2026-02-03

**Authors:** Colton Zapp, Donald M Downer II, Alby Levine, Natori Parker, Mark Zapp, Donald Downer, Larry Levine

**Affiliations:** 1 Research, Fleming Island Surgery Center, Fleming Island, USA; 2 Research, East West Surgery Center, Fleming Island, USA; 3 Anesthesiology, Fleming Island Surgery Center, Fleming Island, USA; 4 Ophthalmology, East West Surgery Center, Fleming Island, USA

**Keywords:** anesthesia, continuing semaglutide, eye surgery, moderate sedation, ozempic, risk of aspiration, semaglutide

## Abstract

Purpose

The purpose of this study was to assess aspiration risk in patients undergoing elective eye surgery with moderate sedation as the anesthetic while taking the glucagon-like peptide type 1 receptor agonist (GLP-1RA) Ozempic formulation of semaglutide.

Setting and design

This retrospective observational study took place in Fleming Island Surgery Center and East West Surgery Center, Fleming Island, Florida.

Methods

Records of patients actively taking the Ozempic formulation of semaglutide and presenting for elective eye surgery from July 1, 2022, through December 31, 2023, were reviewed to determine the incidence of aspiration. The patient risk assessment included body mass index in kg/m2 (BMI), and medical history regarding diabetes mellitus (DM), gastroesophageal reflux disease (GERD), hiatal hernia (HH), and narcotic use. Analysis assessments included the time and dose of the last semaglutide administration (0-7 days, 8-14 days, or >14 days), type and duration of surgery, type of anesthesia or sedation, pre- and post-operative oxygen saturation (SpO2), respiratory problems, and post-operative nausea or vomiting (PONV).

Results

A total of 155 surgical records of patients actively taking semaglutide were identified and reviewed. The BMI range was 21-52; 143 cases had DM (all DM cases were type 2), 58 cases had GERD, and 12 cases had concurrent narcotic use. One hundred fifty-one patients received moderate sedation, two received intravenous propofol with local block, and two underwent general anesthesia. One hundred twenty of the 155 cases had taken semaglutide within one week of the surgery. There were no significant intra- or postoperative respiratory issues, PONV, or significant changes in the postoperative SpO2 in any of the patients.

Conclusion

There were no cases of aspiration or respiratory compromise in any patients who continued semaglutide at the time of their surgical procedure. In this study, continuing semaglutide while undergoing elective eye surgery with only moderate sedation for anesthesia did not pose an aspiration risk, minimized disruption to patient medical routines, and reduced the negative impact of procedure cancellation.

## Introduction

Semaglutide is a glucagon-like peptide type 1 receptor agonist (GLP-1RA). By attaching to GLP receptors in the body, it increases insulin secretion, decreases glucagon secretion, and delays gastric emptying [1]. There are anecdotal reports of retained gastric contents and increased risk of aspiration with deep sedation in patients on this medication [2,3]. This led the American Society of Anesthesiologists (ASA) to publish guidance in June 2023 for patients undergoing elective procedures while taking GLP-1RAs [4]. They recommended that patients on daily dosing hold their GLP-1RA the day of the procedure. For patients on weekly dosing, they recommended holding their GLP-1RA a week before the procedure. No studies served as reference for these recommendations in the setting of elective outpatient eye surgery with moderate sedation for anesthesia. If these medications were not withheld, patients would be treated as if they had residual food in their stomachs. This would mean cancelling their eye surgeries until the appropriate GLP-1RA-free interval had passed. Subsequently, in October 2024, a multispecialty society, including the ASA, issued a new statement stating that most patients (with modifications to NPO status or gastric ultrasound testing) could continue their GLP-1RA before elective surgery [5].

Continued studies of patients undergoing surgical procedures while on GLP-1RA medications have been recommended and should be considered when assessing the risks of surgery while on these medications [6-9]. The purpose of this study is to add to the data on patients undergoing elective eye surgery while on semaglutide.

This was a retrospective review of eye surgeries performed on patients taking the Ozempic formulation of semaglutide. Other formulations were not included as there was only one patient in our original search on the Wegovy formulation, and no cases of compounded semaglutide. Services were provided at two ambulatory surgery centers. Patients in the study were placed into subgroups based on preoperative withholding times of semaglutide prior to their surgery: 0-7 days, 8-14 days, or more than 14 days. The records of these patients were reviewed for documentation of aspiration, respiratory distress, nausea or vomiting, and pre-operative and post-operative SpO2. Most of the surgeries were cataract operations. Most of the cataract patients had their first procedure and then returned two weeks later for their second procedure. Our objective was to determine whether continuing semaglutide increases aspiration risk in elective eye surgery patients with only moderate sedation for anesthesia.

## Materials and methods

A retrospective analysis was conducted on all patients who electively had outpatient eye surgery between July 1, 2022, and December 31, 2023, with Clay Eye Physician and Surgeons in Fleming Island, Florida. HIPAA regulations were followed. Board approval was obtained. Data was reviewed from two separate ambulatory surgery centers (East West Surgery Center and Fleming Island Surgery Center). The same group of ophthalmologists and anesthesia providers delivered patient care at both surgery centers.

Patients were screened to include those who were actively using weekly semaglutide prior to their eye surgery. All patients having surgery in each of the surgery centers were searched through a secure electronic health record for the inclusion of semaglutide as part of their medication regimen. This study did not include patients using any other formulations or other GLP-1RAs. Our original search only found one case of the Wegovy formulation of semaglutide and no cases of compounded semaglutide. This one case was not included in our study. A total of 9328 eye surgery cases were screened. This generated data from 88 patients (n=155 surgeries) based on the inclusion criteria. Patient charts were reviewed for demographic information, BMI, date and dose of last administered semaglutide, presence of diabetes mellitus (DM; all patients had type 2 DM), and use of any narcotics preoperatively. In addition, patient records were reviewed for a gastrointestinal history of hiatal hernia (HH), or gastroesophageal reflux disease (GERD). The types and lengths of eye surgeries were recorded. Descriptive statistical analysis was included and calculated through Excel spreadsheets (Microsoft, Redmond, Washington).

All patients had nothing by mouth for a minimum of eight hours for solids and two hours for clear liquids prior to the surgery. The types of anesthesia were moderate sedation, deep sedation with intravenous propofol and retrobulbar block, or general anesthesia. Moderate sedation cases were administered intravenous midazolam and fentanyl. Doses of anesthetic agents used during the surgery were documented. Detailed postoperative reports were reviewed to document any nausea, vomiting, or respiratory distress. Preoperative and postoperative SpO2, as measured by pulse oximetry, were recorded.

The patients were categorized by the last date of semaglutide administration before surgery: 0-7 days, 8-14 days, and greater than 14 days. These groups were chosen as there was some ambiguity on the meaning of withholding GlP-1RAs. It is unclear if the ASA's recommendation of holding GLP-1RAs a week prior to the procedure meant it was acceptable for them to take the medication seven days before or whether they should be off for at least 14 days. Some physicians interpret taking these medications seven days before as not really holding the medication. If the patient was taking semaglutide but the time of the last dose was not clearly delineated, it was classified in a separate unknown category. Analysis was done to determine if there was any relationship between the use of semaglutide preoperatively and subsequent respiratory complications indicative of aspiration in an outpatient eye surgery setting. The study also analyzed any complications in patients with co-existing aspiration risk factors that included increased BMI, DM, GERD, HH, and the use of narcotics preoperatively.

## Results

The baseline data is found in Table 1.

**Table 1:** Baseline data This data contain the average age, BMI, and dose of semaglutide.  It also includes the number of patients based on gender, presence of diabetes, presence of gastroesophageal reflux disease, and preoperative narcotic use. DM - diabetes mellitus; GERD - gastroesophageal reflux disease

Withholding time	0-7 Days	8-14 Days	> 14 Days	Unknown
Age	65.6	70	66.2	69.3
Male	44	2	1	13
Female	76	3	3	13
Average BMI	34	32	37	35
DM	113	5	3	22
GERD	45	1	0	8
Semaglutide dose mg	0.8	1.8	1.2	1.1
Preop narcotic #	8	0	2	2

The majority of patients were female (61.3%). In the analysis of 155 surgical procedures on 88 patients, there were no aspirations. Nausea was minimal 1/155 (0.6%), and there was no vomiting. Patients' BMIs varied from 21 to 52. In 143/155 (92.3%) of the procedures, the patients had DM, and 12/155 (7.7%) did not have DM. All of the patients with DM had type 2 DM. Twenty-nine of the patients (totaling 56 procedures) were also on insulin for their DM.

**Figure 1:**
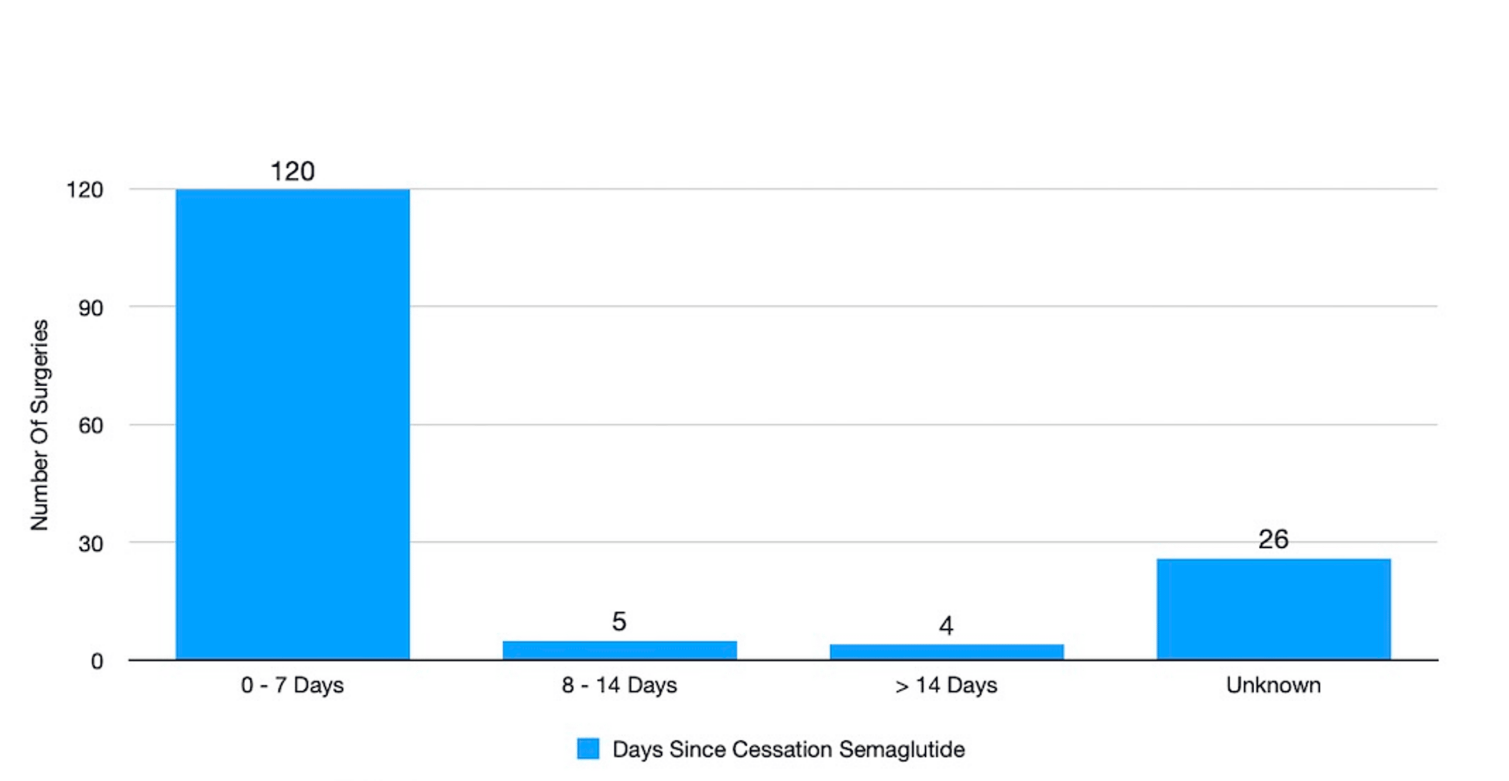
Timing of Ozempic cessation prior to surgery The majority of patients in our study did not follow American Society of Anesthesiologist guidance regarding cessation of semaglutide, receiving it within seven days of their surgeries. The unknown category represents patients who were on semaglutide, but the time of last administration was unclear.

In 120 (77.4%) of the surgeries, the patients continued semaglutide, administering the last dose within seven days of the operation (Figure 1).

**Figure 2:**
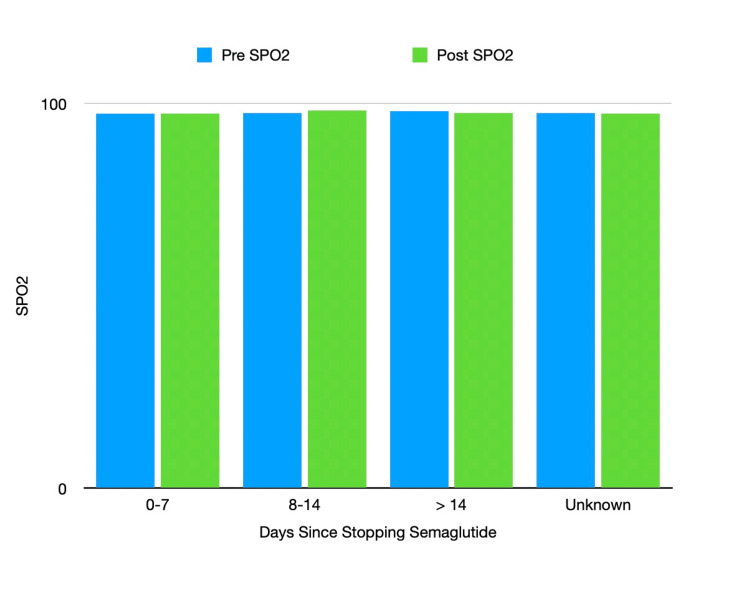
Preoperative and postoperative SpO2 vs days since stopping semaglutide Preoperative and post-operative SpO2 for each duration of cessation of semaglutide for our analyzed categories. The unknown category represents the group where patients were taking semaglutide, but the time of last administration was unclear.

In this group, there were no aspirations and one occurrence of nausea without vomiting. The average preoperative SpO2 was 97.4, and the average postoperative SpO2 was 97.3% for these patients (Figure 2, Table 2).

**Table 2:** Pre and postoperative SpO2 in each study group with descriptive statistics

Statistic	Pre 0-7 days	Post 0-7 days	Pre 8-14 days	Post 8-14 days	Pre >14 days	Post >14 days	Pre unknown	Post unknown
Mean	97.4	97.3	97.6	98.2	98	97.5	97.6	97.3
Standard error	0.15	0.15	0.40	0.58	0	0.50	0.32	0.34
Median	97.5	97	97	98	98	98	98	97.5
Mode	97	98	97	97	98	98	98	98
Standard deviation	1.69	1.67	0.89	1.30	0	1	1.63	1.72
Sample variance	2.84	2.78	0.80	1.70	0	1	2.65	2.96
Minimum	92	92	97	97	98	96	95	94
Maximum	100	100	99	100	98	98	100	100

The majority, 113/120 (94.2%), had DM. The average BMI for this group was 33.

In 5/155 (3.2%) of the surgeries, semaglutide was withheld for a period of 8-14 days prior to surgery (Figure 1). There were no aspirations, nausea, or vomiting in this group. The average preoperative SpO2 was 97.6%, and the average postoperative SpO2 was 98.2% (Figure 2, Table 2). Five out of five (100%) of this group had DM. The average BMI was 34.

In 4/155 (2.6%) of these surgeries, semaglutide was withheld for a period greater than 14 days prior to surgery (Figure 1). There were no aspirations, nausea, or vomiting in this group. The average preoperative SpO2 was 98.0%, and the average post-operative SpO2 was 97.5% (Figure 2, Table 2). Three out of four (75%) of the surgeries involved diabetic patients. The average BMI was 37 in this group.

In 26/155 (16.8%) of the surgeries, the last administered date of semaglutide was unclear in the medical record. These were classified as unknown. There were no aspirations, nausea, or vomiting in this group. The average pre-operative SpO2 was 97.6%, and the average post-operative SpO2 was 97.3% Figure 2, Table 2). The majority, 22/24 (91.7%) of the surgeries involved patients with DM. The average BMI for this group was 35.

Excluding the unknown group, 120/129 (93.0%) of the cases did not meet the ASA guidance of stopping semaglutide one week before surgery. There were no aspirations, 1/120 (0.8%) case of nausea, and no vomiting was recorded in this group.

A review of the medication records documented that 12/155 (7.7%) of the patients were taking narcotics before surgery. There were six surgeries in which the patients were taking hydrocodone and six surgeries in which the patients were taking oxycodone concurrently with semaglutide at the time of their eye surgery. There were no aspirations in any of these patients.

**Figure 3:**
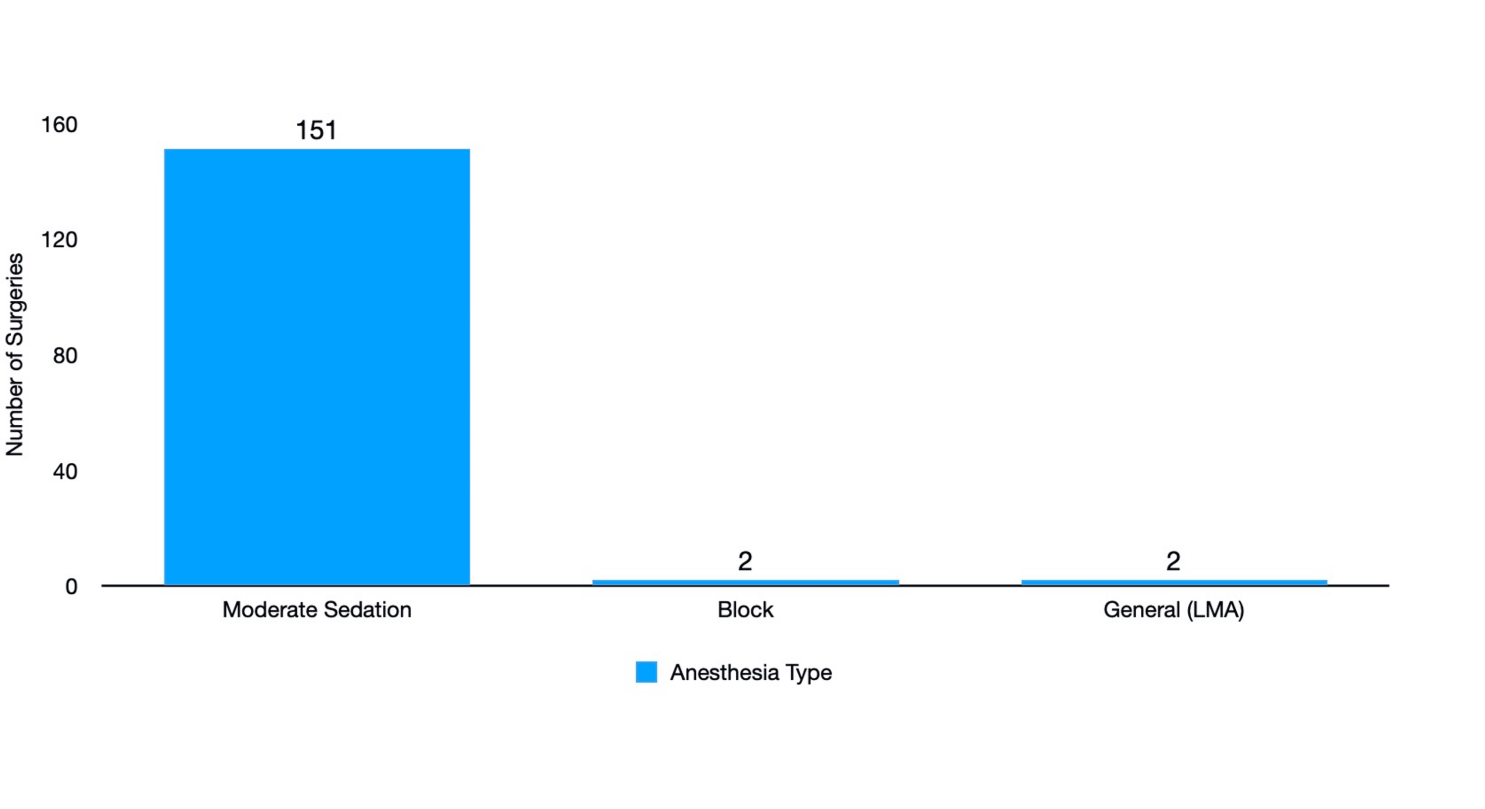
Distribution of anesthesia types Most anesthesia types were moderate sedation with the administration of fentanyl and versed. Block cases included administering propofol for sedation for a retrobulbar block, followed by administering moderate sedation during the surgery. General anesthetic cases involved the administration of propofol for induction, placing an LMA, and administering sevoflurane for maintenance of anesthesia. LMA - laryngeal mask airway

Moderate sedation was used in 151/155 (97.4%) of the cases. Deep sedation with propofol and a retrobulbar block was used in 2/155 (1.3%) cases, and general anesthesia with laryngeal mask airway (LMA) was used in 2/155 (1.3%) of the cases (Figure 3).

**Figure 4:**
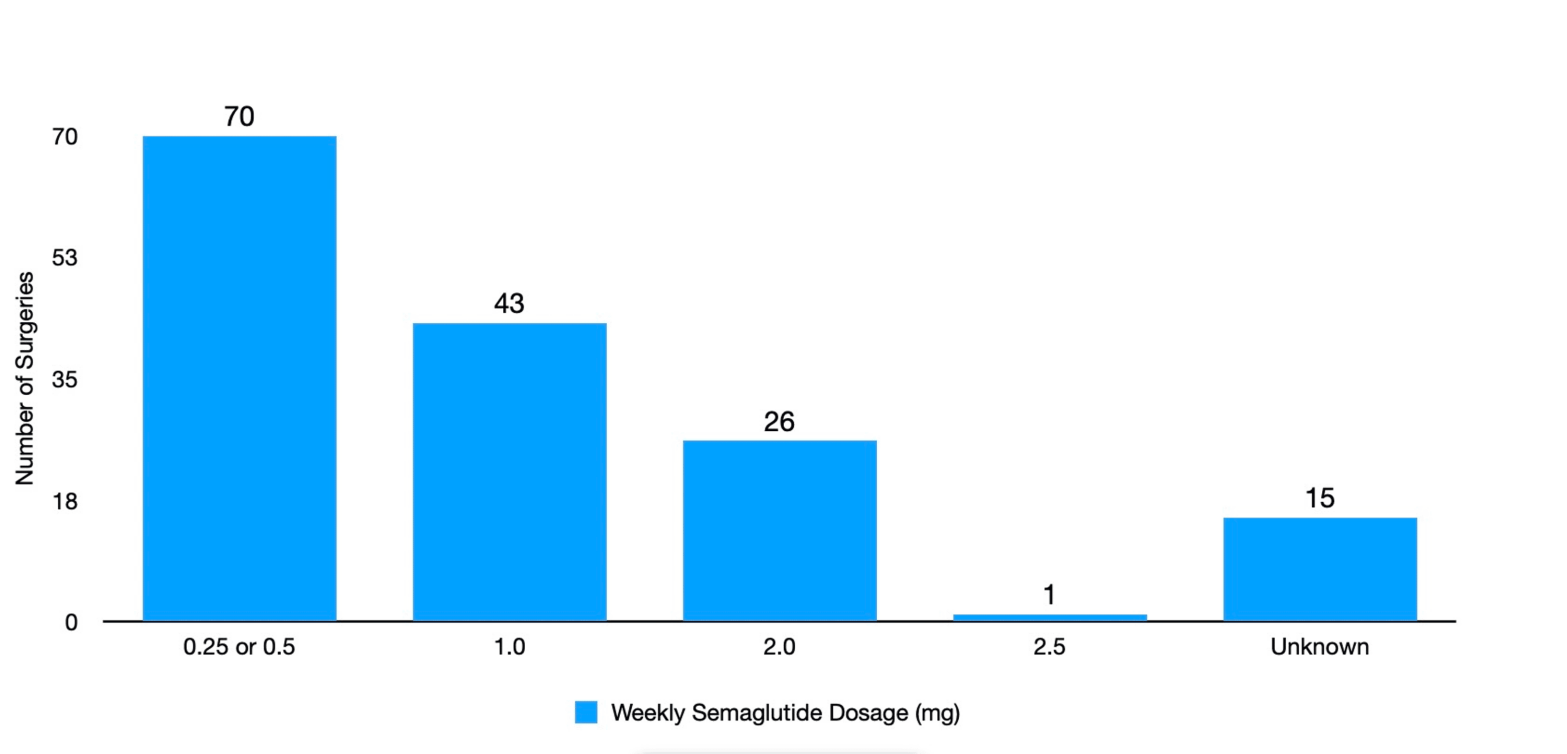
Distribution of semaglutide dosages administered during surgeries The most common dosages of semaglutide administered were lower dosages. The least common dosage administered was 2.5 mg, the highest dosage in our study. In 15 patients, the dosage of semaglutide was unclear. This was categorized as the unknown group.

There were no aspirations in this group. The distribution of GLP-1RA weekly semaglutide dosages revealed that the most frequent administration was 0.25 or 0.5 mg (45.2%), followed by 1 mg (27.7%), 2 mg (16.8%), and 2.5 mg (0.6%). In 15/155 (9.7%) of cases, dosage information was unavailable in the patient's history (Figure 4).

**Figure 5:**
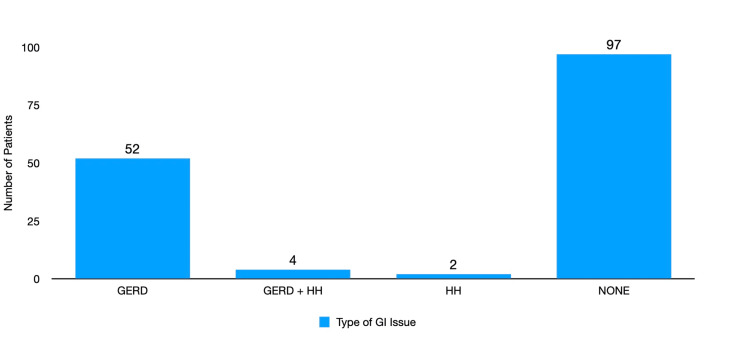
Incidence of gastrointestinal disease in patients taking semaglutide GERD and HH may increase the risk of aspiration. The majority (62.6%) of patients did not have a history of GERD or HH, but a minority (37.4%) did have these diagnoses. GERD - gastroesophageal reflux disease; HH - hiatal hernia

There were 58/155 (37.4%) cases performed on patients with a history of gastrointestinal issues (52/155 GERD, 4/155 GERD with HH, and 2/155 HH alone). Ninety-seven of 155 (62.6%) of the patients had no history of gastrointestinal issues (Figure 5). 

There were no documented aspirations in patients with a history of GERD or HH taking semaglutide.

Anxiolytic sedation included 1 mg of midazolam (17.4%), 2 mg of midazolam (67.7%), and greater than 2 mg of midazolam (14.1%). Narcotic sedation included 50 µg of fentanyl (64.5%) or 75-100 µg of fentanyl (23.2%). Some cases (12.3%) did not receive fentanyl.

Surgical durations varied, with cataract surgeries having an average time of 9.21 ± 5.9 and 8.63 ± 4.74 minutes for the left and right eye, respectively (Table 3).

**Table 3:** Statistics on right and left cataract surgery times in minutes

Surgery time statistic	Right eye	Left eye
Mean	8.67	9.40
Standard error	0.54	0.51
Median	7	8
Mode	6	5
Standard deviation	4.74	6.37
Sample variance	22.50	40.53
Kurtosis	6.10	8.04
Skewness	1.97	2.47
Minimum	2	2
Maximum	31	41
Largest (1)	31	41
Smallest (1)	2	2
Confidence Level (95.0%)	1.07	1.02

More complex procedures showed greater variability. Specific average durations included cataract plus minimally invasive glaucoma surgery (MIGS) in the left eye at 27 minutes, cataract plus MIGS in the right eye at 12 minutes, tube shunt placement at 22 minutes, pterygium excision with graft at 41 minutes, and pars plana vitrectomy at 38 minutes. However, regardless of the length or type of procedure, amount of sedation, gastrointestinal history, narcotic use, DM, or BMI, none of the 155 cases resulted in aspirations.

## Discussion

The use of GLP-1RAs continues to increase. Currently, about nine million Americans are taking these medications. The latest Kaiser Family Foundation Health Tracking poll shows that about one in eight adults (12%) have taken a GLP-1RA, and 6% are currently taking them [10]. According to estimates from the US Centers for Disease Control and Prevention, about 15% of adults in the United States have diabetes, and more than 40% have obesity [11]. Other benefits of GLP-1RAs include improvements in cholesterol profiles, steatohepatitis, neurodegenerative diseases, and sleep apnea [12-15]. There is also reduced cardiovascular death, heart attack, stroke, chronic kidney disease in diabetics, and cravings in substance abuse disorders [16-18]. As such, a presumptive growth in patients taking these medications can be expected.

Exogenous GLP-1RA relaxes the proximal stomach in a dose-dependent manner, reduces antral and duodenal motility, and increases pyloric tone in both the fasted and fed states [19]. A retrospective study on patients taking GLP-1RAs who had esophagogastroduodenoscopy (EGD) between 2019 and 2023 found a four-fold increase in retention of gastric contents and four-fold higher rates of aborted EGD (p<0.0001) [20]. Aspiration may be as high as 3-5% in patients undergoing endoscopy procedures under deep sedation [21-22]. Other risk factors for aspiration under anesthesia include the presence of DM or GERD, preoperative narcotic use, and obesity. Despite the high incidence of DM and obesity in our study, there were no aspirations (even when semaglutide was continued).

While aspiration may be a concern for endoscopy procedures, it may not be a concern for eye surgery with only moderate sedation for anesthesia. In fact, there was a 200-patient study on patients taking oral sedation for cataract procedures without restrictions to food intake, claiming happier patients and no aspirations [23]. There was also an 11,218-case study on non-fasted cataract patients having oral midazolam and low-dose propofol (0.25mg/kg) injections as needed with no documented aspirations [24]. A study on 5125 cataract patients without restrictions to PO status and about 50% receiving moderate sedation demonstrated no aspirations [25]. Therefore, the original ASA guidance may not apply to eye surgery patients receiving only moderate sedation who can maintain oral reflexes, do not necessarily require airway instrumentation, and are not subject to increased abdominal pressure during the procedure.

Discontinuing GLP-1RA medications may be associated with other problems, including hyperglycemia, worsening cholesterol profile, possible weight gain, stress, demoralizing effects, and health care delays [26]. In addition, cancelling eye surgeries when the patient remains on these medications adds significant inconvenience, disruption, and potential costs to the patient and their families. Surgical cancellations are also associated with interrupted operating room efficiency and increased health facility expenses.

This study demonstrated no aspirations when analyzing over 18 months of data with a search of over 9000 patients to find 155 cases of patients actively taking semaglutide. If we had larger numbers of patients, we might have been able to include inferential statistics, but continued study is difficult with current guidance. This study was performed at two private practice surgery centers, limiting external validity, but it should be easily reproducible. Other limitations of this study include that it was a retrospective chart review. This introduces the possibilities of observation, selection, information, and reviewer biases and confounding. We relied on documentation of the records to be accurate, but information and reviewer biases could exist. The study was performed at outpatient centers with efficient surgeons and anesthesia providers. This introduces selection bias and should not be applied to all surgical settings or procedures with longer surgical times. We attempted to limit biases by analyzing objective findings like documented nausea or vomiting and comparison of pre- and postoperative SPO2, but more subtle or silent aspirations might have occurred. However, if aspirations are silent or subtle, clinical significance is doubtful. Gastric aspiration is believed to occur when patients with residual gastric contents receive sedation, these gastric contents spread through the esophageal lumen, and the contents enter the lungs, causing a chemical pneumonitis and possible pneumonia. There may be other requirements for this to occur, including emergency surgery, abdominal surgery, abdominal pressure, airway manipulation, or other coexisting pathology that did not occur in our population [20-22,27]. It is also possible that preoperative patients who had nausea or were not feeling well from taking semaglutide may not have even presented for surgery, eliminating a potential higher-risk group. While this study contains some cases with deep sedation or general anesthesia, these numbers are too few to determine any consideration of continuing GLP-1 RAs. Furthermore, we still recommend following guidance and requesting patients to stop GLP-1 RAs before elective surgery. However, despite screening, patients still present to our centers while still on these medications. Here, a symptom-based approach might be an option, with a gastric ultrasound if available. In addition, our data were restricted to the Ozempic formulation of semaglutide and may not apply to other formulations or other GLP-1 RAs.

It is estimated that around four million cataract surgeries are performed in the US annually, and more than 26 million are performed worldwide [28]. These are usually performed in an outpatient setting under no, mild, or moderate sedation and with administration of topical anesthetic drops. Our retrospective analysis of patients taking semaglutide while undergoing eye surgery to assess for increased risk of aspiration found no evidence of aspiration or respiratory distress. Following current ASA guidance (withholding GLP1-RAs) does not allow continued study of aspiration risk for this population of cataract patients. This study was specifically designed to analyze patients before ASA guidance was established. Therefore, it significantly adds to the GLP-1RA literature.

## Conclusions

Continuing semaglutide in patients undergoing eye surgery with only moderate sedation appears to have a low risk of aspiration and did not demonstrate any risk in our study. Over an 18-month study period, there was a zero percent incidence of aspiration in patients taking semaglutide. This finding adds information to the updated ASA guidance statement on GLP-1RA medications. This study would apply particularly to cataract patients, in which the procedure is relatively short, and the sedation level is moderate.
